# Pattern of liver injury in adult patients with COVID-19: a retrospective analysis of 105 patients

**DOI:** 10.1186/s40779-020-00256-6

**Published:** 2020-06-07

**Authors:** Qi Wang, Hong Zhao, Li-Gai Liu, Yan-Bin Wang, Ting Zhang, Ming-Hui Li, Yan-Li Xu, Gui-Ju Gao, Hao-Feng Xiong, Ying Fan, Ying Cao, Rui Ding, Jing-Jing Wang, Cheng Cheng, Wen Xie

**Affiliations:** 1 Center of Liver Diseases, Beijing Ditan Hospital, Capital Medical University, Beijing, 100,015 China; 2 Beijing Key Laboratory of Emerging Infectious Diseases, Beijing, 100,015 China; 3 Clinical and Research Center of Infectious Diseases, Beijing Ditan Hospital, Capital Medical University, Beijing, 100,015 China; 4 Department of Critical Care Medicine, Beijing Ditan Hospital, Capital Medical University, Beijing, 100,015 China

**Keywords:** Coronavirus disease-2019, Liver function, Dynamic change

## Abstract

**Background:**

Recent studies reported that patients with coronavirus disease-2019 (COVID-19) might have liver injury. However, few data on the combined analysis and change patterns of alanine aminotransferase (ALT), aspartate aminotransferase (AST) and total bilirubin (TBil) have been shown.

**Methods:**

This is a single-center retrospective study. A total of 105 adult patients hospitalized for confirmed COVID-19 in Beijing Ditan Hospital between January 12, and March 17, 2020 were included, and divided into mild group (*n* = 79) and severe group(*n* = 26). We compared liver functional test results between the two groups. Category of ALT change during the disease course was also examined.

**Results:**

56.2% (59/105) of the patients had unnormal ALT, AST, or total TBil throughout the course of the disease, but in 91.4% (96/105) cases the level of ALT, AST or TBil ≤3 fold of the upper limit of normal reference range (ULN). The overall distribution of ALT, AST, and TBil were all significantly difference between mild and severe group (*P* <  0.05). The percentage of the patients with elevated both ALT and AST was 12.7% (10/79) in mild cases vs. 46.2% (12/26) in severe cases (*P* = 0.001). 34.6% (9/26) severe group patients started to have abnormal ALT after admission, and 73.3% (77/105) of all patients had normal ALT before discharge.

**Conclusions:**

Elevated liver function index is very common in patients with COVID-19 infection, and the level were less than 3 × ULN, but most are reversible. The abnormality of 2 or more indexes is low in the patients with COVID-19, but it is more likely to occur in the severe group.

## Background

Core clinical features in patients with coronavirus disease-2019 (COVID-19) include fever, dry cough and asthenia. Patients with severe illness often experience dyspnea and/or hypoxemia within one week after symptom onset, and the mortality rate is about 1–3% [1]. Liver biochemical abnormalities have been documented in COVID-19 patients, and typically include mild elevations of alanine aminotransferase (ALT) and aspartate aminotransferase (AST), ranging from 14 to 53% [1–4]. Patients with severe illness, and particularly those requiring intensive care unit (ICU) admission, tend to have higher rate of transaminase elevation than in those with mild to moderate illness [4, 5]. No studies have reported the incidence of concurrent elevation of serum transaminases and total bilirubin (TBil) in patients with COVID-19. Also, few studies examined the dynamic change of liver function throughout the course of COVID-19.

## Methods

### Study design

This is a single-center retrospective study. Medical records were screened to identify adult patients receiving treatment for confirmed COVID-19 at Beijing Ditan Hospital, Capital Medical University, from January 12, 2020 to March 17, 2020. The cases were divided into mild group (mild and ordinary type, *n* = 79) vs. severe group (severe and critical type, *n* = 26) according to their condition [6]. This study was approved by the Ethics Committee of Beijing Ditan Hospital, Capital Medical University (#2020–010-01).

### Patient selection

For inclusion in the analysis, patients must be ≥18 years of age. The diagnosis and classification (into mild vs. severe cases) of COVID-19 was based on the “New Coronavirus Pneumonia Diagnosis and Treatment Plan (Trial Edition 4–6)” published by the Chinese Health and Health Council [6]. Mild type was defined as lack of signs for pneumonia based on image results. Ordinary type was defined as having fever and respiratory symptoms radiologic signs of pneumonia. Severe type was defined as the presence of any of the following: respiratory distress (respiratory rate > 30 times/min), oxygen saturation SpO_2_ (resting state) ≤ 93%, abnormal blood gas analysis: partial arterial oxygen pressure (PaO_2_)/fraction of inspired oxygen (FiO_2_) ≤ 300 mmHg. Critical type was defined as the presence of any of the following: respiratory failure that requires mechanical ventilation, shock, accompanied by other organ failure that needs ICU admission. COVID-19 was based on PCR for the SARS-CoV-2 gene using nasal or pharyngeal swabs taken prior to admission. All patients received at least one chest CT scan after admission. The criteria for discharge from hospital included: Normal body temperature for at least 3 days; resolution of respiratory symptoms; chest CT showing improvement in lung inflammation; two-negative RT-PCR results for SARS-CoV2 respiratory samples at least 24 h apart [6].

Exclusion criteria: 1) Patients who were still hospitalized in Beijing Ditan Hospital, Capital Medical University until March 17, 2020. 2) The patients did not have complete medical history, and in particular, liver function tests were not performed during hospitalization.

### Data collection

The following information was extracted from the medical records: sex, age, alcohol use, time of onset, hospital stay and existing diseases. Standard liver blood chemistry was conducted at admission as well as during hospitalization. Some patients also underwent abdominal ultrasound examination. The normal reference ranges were: 1) ALT: male 9.0–50.0 U/L, female 7.0–40.0 U/L; 2) AST: male 15.0–40.0 U/L, female 13.0–35.0 U/L; 3) TBil: 0-18.8 μmol/L; 4) albumin (ALB): 40.0–55.0 g/L; 5) cholinesterase (CHE): 4000–11,000 U/L.

### Statistical analysis

All statistical analyses were conducted using SPSS (Version 17.0). Categorical variables were presented as percentages, and continuous variables as means + standard deviations (SD) or medians and inter-quartile ranges (IQR, 25-75th). Comparisons between groups were made by using chi-square test for mortality or differences in categorical variables and Student’s *t*-test or Wilcoxon rank sum test for continuous variables, as appropriate. *P* <  0.05 was considered statistically significant.

## Results

### Patient selection process

**Fig. 1:**
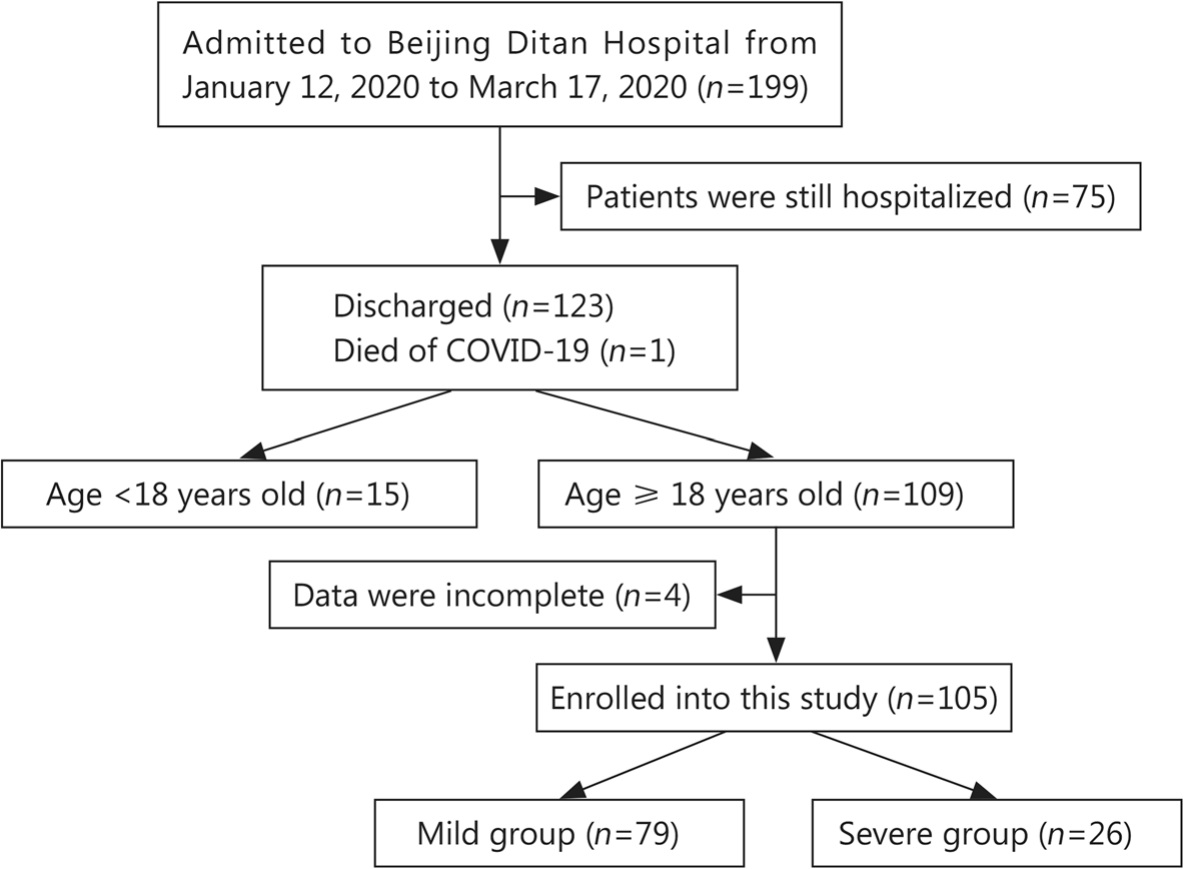
The flow of patient enrollment. From January 12, 2020 to March 17, 2020, 199 patients were admitted to Beijing Ditan Hospital, Capital Medical University. One hundred twenty three patients were discharged and 1 patient was died of COVID-19. One hundred nine patients were old than 18 years. But data of 4 patients were incomplete. At last, 105 patients were enrolled into this study

A total of 105 patients were included in this study, accounting for 52.8% of the patients receiving treatment for COVID-19 at Beijing Ditan Hospital, Capital Medical University from January 12, 2020 to March 17, 2020. Patient selection for inclusion in the analysis is shown in Fig. 1.

### Clinical characteristics at admission

The results of clinical characteristics at admission are presented in Table 1. The median age was 45.0 years (IQR: 33.5, 59.5; range: 18 to 92) in the entire cohort, 41.0 years (IQR: 33.0, 56.0) in mild cases (*n* = 79) and 59.0 years (48.5, 69.8) in severe cases (*n* = 26) (*P < 0.001*). The male-female ratio was 1:1 in mild cases and 2.2:1 in severe cases (*P* = 0.073). The time from symptom onset to hospital admission was 4.0 days in the mild cases vs. 7.0 days in severe cases (*P* = 0.005). The median hospital stay was 22.0 days in the entire cohort, 20.0 days in mild cases vs. 31.5 days in severe cases (*P* = 0.001).

**Table 1:** Clinical characteristic of the patients with COVID-19 at admission

Item	Overall	Mild group	Severe group	*P*-value
Male [n (%)]	56/105 (53.3)	38/79 (48.1)	18/26 (69.2)	0.073
Age (year, IQR)	45.0 [33.5, 59.5] (*n* = 105)	41.0 [33.0, 56.0] (*n* = 79)	59.0 [48.5, 69.8] (*n* = 26)	< 0.001
Disease duration (d, IQR)	5.0 [3.0, 8.0] (*n* = 105)	4.0 [2.0, 7.0] (*n* = 79)	7.0 [5.0, 10.25] (*n* = 26)	0.005
Hospital stay (d, IQR)	22.0 [17.0, 31.5] (*n* = 105)	20.0 [16.0, 28.0] (*n* = 79)	31.5 [22.5, 35.5] (*n* = 26)	0.001
Fatty liver by ultrasound [n (%)]	37/89 (41.6)	26/65 (40.0)	11/24 (45.8)	0.636
Hypertension [n (%)]	15/105 (14.3)	9/79 (11.4)	6/26 (23.1)	0.190
Diabetes [n (%)]	6/105 (5.7)	3/79 (3.8)	3/26 (11.5)	0.160
HBsAg (+) [n (%)]	0/22 (0)	0/14 (0)	0/8 (0)	–
Anti-HCV (+) [n (%)]	1/22 (0)	0/14 (0)	1/8 (12.5)	0.364
Heavy alcohol use [n (%)]	1/105 (1.0)	1/79 (1.3)	0/26 (0)	1.000
ALT (U/L, IQR)	23.5 [14.0, 36.0] (*n* = 105)	22.0 [14.0, 34.5] (*n* = 79)	27.8 [18.8, 38.0] (*n* = 26)	0.088
< 1 × ULN [n (%)]	88 (83.8)	67 (84.8)	21 (80.8)	0.482
1–2 × ULN [n (%)]	13 (12.4)	10 (12.7)	3 (11.5)	
≥2 × ULN [n (%)]	4 (3.8)	2 (2.5)	2 (7.7)	
AST (U/L, IQR)	24.2 [19.7, 34.8] (*n* = 50)	22.0 [18.4, 31.7] (*n* = 39)	46.3 [25.5, 54.3] (*n* = 11)	< 0.001
< 1 × ULN [n (%)]	41 (82.0)	37 (94.9)	4 (36.4)	< 0.001
1–2 × ULN [n (%)]	8 (16.0)	2 (5.1)	6 (54.5)	
≥2 × ULN [n (%)]	1 (2.0)	0 (0)	1(9.1)	
TBil (umol/L, IQR)	10.2 [7.4, 12.9] (*n* = 50)	10.0 [7.1, 12.9] (*n* = 39)	10.6 [8.3, 12.9] (*n* = 11)	0.535
< 1 × ULN [n (%)]	48 (96.0)	38 (97.4)	10 (90.9)	0.395
1–2 × ULN [n (%)]	2 (4.0)	1 (2.6)	1 (9.1)	
≥2 × ULN [n (%)]	0 (0)	0 (0)	0 (0)	
CHE (U/L, IQR)	7490 [6801, 9527] (*n* = 50)	6517 [6843, 9682] (*n* = 39)	6972 [4893, 8459] (*n* = 11)	0.137
ALB (g/L, IQR)	41.6 [37.9, 44.7] (*n* = 49)	42.0 [38.7, 45.5] (*n* = 39)	37.2 [34.2, 41.8] (*n* = 10)	0.012

*IQR* interquartile range, *ALT* alanine aminotransferase, *AST* aspartate aminotransferase, *TBil* total bilirubin. *ALB* albumin, *CHE* cholinesterase

A total of 89 out of 105 patients underwent abdominal ultrasound scans for fatty liver. Fatty liver was detected in 40.0% of the mild cases vs. 45.8% in severe cases (*P* = 0.636). One patient had extended history of heavy alcohol use (ethanol consumption at > 40 g/d). All patients denied a history of chronic hepatitis B or chronic hepatitis C. HBsAg, anti-HCV, anti-HIV, and syphilis-specific antibodies were tested in 22 patients; the results showed positive anti-HCV but no HCV RNA in one patient. Among the 37 patients with fatty liver, 11 (29.7%) had elevated ALT; 6 (21.6%) were mild cases and 5 (13.5%) were severe cases. In 9 out of the 11 cases, ALT elevation was < 2 × ULN (upper limit of normal reference range); the highest ALT level was 129.9 U/L. We did not perform statistical analysis due to the small sample size.

The analysis results of liver functions at admission are also presented in Table 1. Seventeen out of 105 patients (16.2%) elevated ALT. The median ALT level was 22.0 U/L in mild cases vs. 27.8 U/L in severe cases (*P* = 0.088). However, there was no difference in the distribution of ALT at different levels between the two groups (*P* = 0.482). The highest ALT was 357.0 U/L, which was appeared on a male mild group patient. AST, TBil, CHE, and ALB levels were available in 50 patients only. The median AST was 22.0 U/L in mild cases vs. 46.3 U/L in severe cases (*P* < 0.001). The rate of isolated AST elevation was higher at 63.6% in severe cases vs. 5.1% in mild cases (P < 0.001). AST was elevated at ≥2 × ULN in one severe cases. The median TBil was 10.0 μmol/L in mild cases vs. 10.6 μmol/L in severe cases (*p* = 0.535). Among the 50 patients with TBil, only 2 cases had elevated TBil (one patient in every group, and both below 2 × ULN). CHE analysis did not show statistically significant difference between the two groups (*p* = 0.137). A total of 22 (30.0%) patients had elevated ALT or AST or TBil, including 14 (17.7%) mild cases and 8(30.8%) severe cases (*P* = 0.156). The median ALB was 42.0 g/L in mild cases vs. 37.2 g/L in severe cases (*P* = 0.012).

### Analysis of liver function during hospitalization

**Fig. 2:**
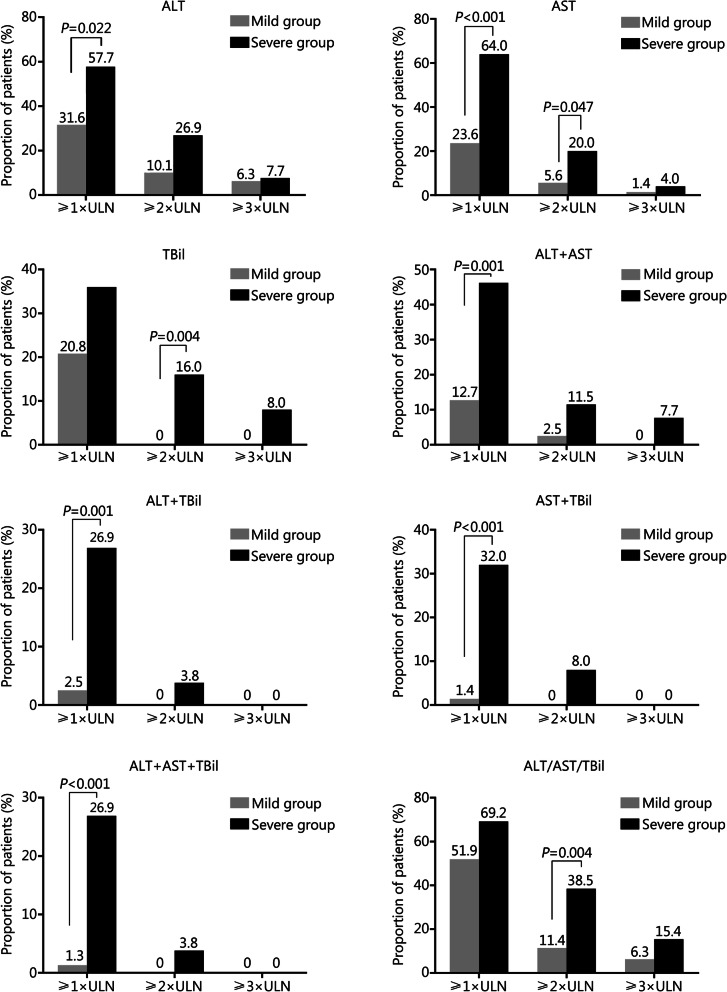
Single or combination analysis of indexes during hospitalization in mild vs. severe cases using different cut-off values. Using the cut-off value as 1, 2, or 3 × ULN, respectively, the differences of the overall distribution and the abnormal rates of liver function indexes between the two groups were analyzed. Most of the elevated liver function index were less than 3 × ULN. The abnormality of 2 or more indexes is low in the patients with COVID-19, but it is more likely to occur in the severe group

We compared the differences of the overall distribution and the abnormal rates of liver function indexes at different cut-off values as 1, 2, or 3 × ULN, respectively, between the two groups (see Table 2 and Fig. 2). One critically ill patient died within the study period. A total of 508 ALT measurements, 383 AST measurements, and 383 TBil measurements during hospitalization were available for analysis. The highest was 357.0 U/L for ALT, 156.3 U/L for AST, and 102.9 μmol/L for TBil. It was showed that the overall distribution of ALT, AST, and TBil were all significantly difference between mild and severe group (*P* < 0.05).

**Table 2:** Liver function of the patients with COVID-19 during hospitalization [n (%)].

Item	Overall	Mild group	Severe group	P-value
ALT	(*n* = 105)	(*n* = 79)	(*n* = 26)	0.026
< 1 × ULN	65 (61.9)	54 (68.4)	11 (42.3)	
1–2 × ULN	25 (23.8)	17 (21.5)	8 (30.8)	
2–3 × ULN	8 (7.6)	3 (3.8)	5 (19.2)	
≥3 × ULN	7 (6.7)	5 (6.3)	2 (7.7)	
AST	(*n* = 97)	(*n* = 72)	(*n* = 25)	0.001
< 1 × ULN	64 (66.0)	55 (76.4)	9 (36.0)	
1–2 × ULN	24 (24.7)	13 (18.1)	11 (44.0)	
2–3 × ULN	7 (7.2)	3 (4.2)	4 (16.0)	
≥3 × ULN	2 (2.1)	1 (1.4)	1 (4.0)	
TBil	(*n* = 97)	(*n* = 72)	(*n* = 25)	0.010
< 1 × ULN	73 (75.3)	57 (79.2)	16 (64.0)	
1–2 × ULN	20 (20.6)	15 (20.8)	5 (20.0)	
2–3 × ULN	2 (2.1)	0 (0)	2 (8.0)	
≥3 × ULN	2 (2.1)	0 (0)	2 (8.0)	
ALT + AST	(*n* = 105)	(*n* = 79)	(*n* = 26)	0.001
< 1 × ULN	83 (79.0)	69 (87.3)	14 (53.8)	
1–2 × ULN	17 (16.2)	8 (10.1)	9 (34.6)	
2–3 × ULN	3 (2.9)	2 (2.5)	1 (3.9)	
≥3 × ULN	2 (1.9)	0 (0)	2 (7.7)	
ALT + TBil ^a^	(*n* = 105)	(*n* = 79)	(*n* = 26)	0.001
< 1 × ULN	96 (91.4)	77 (97.5)	19 (73.1)	
1–2 × ULN	8 (7.6)	2 (2.5)	6 (23.1)	
2–3 × ULN	1 (1.0)	0 (0)	1 (3.8)	
AST + TBil ^a^	(*n* = 97)	(*n* = 72)	(*n* = 25)	< 0.001
< 1 × ULN	88 (90.7)	71 (98.6)	17 (68.0)	
1–2 × ULN	7 (7.2)	1 (1.4)	6 (24.0)	
2–3 × ULN	2 (2.1)	0 (0)	2 (8.0)	
ALT + AST + TBil ^a^	(*n* = 105)	(*n* = 79)	(*n* = 26)	< 0.001
< 1 × ULN	97 (92.4)	78 (98.7)	19 (73.1)	
1–2 × ULN	7 (6.7)	1 (1.3)	6 (23.1)	
2–3 × ULN	1 (1.0)	0 (0)	1 (3.8)	
ALT/AST/TBil	(*n* = 105)	(*n* = 79)	(*n* = 26)	0.017
< 1 × ULN	46 (43.8)	38 (48.1)	8 (30.8)	
1–2 × ULN	40 (38.1)	32 (40.5)	8 (30.8)	
2–3 × ULN	10 (9.5)	4 (5.1)	6 (23.1)	
≥3 × ULN	9 (8.6)	5 (6.3)	4 (15.4)	

*ALT* alanine aminotransferase, *AST* aspartate aminotransferase, *TBil* total bilirubin

^a^: In this category, the number of cases that meet the index level > 3 × ULN is 0

In the single index analysis, the percentage of the patients having only one abnormal index was 56.2% in the entire cohort, 51.9% in mild cases vs. 69.2% in severe cases (*P* = 0.122). Nineteen (18.1%) patients had elevated ALT, AST or TBil levels ≥2 × ULN: 10 in severe cases and 9 in mild cases (*P* = 0.004).

In the combined analysis, ALT or AST associated with TBil and elevation of all 3 indices were more common in severe cases (*P* < 0.001 or *P* = 0.001). The proportion of elevated TBil with elevated AST was 9.3%, and TBil with ALT was 8.6% (*P* = 1.000). A total of 8 patients had elevation in all 3 indices: seven in severe cases vs. only one in mild cases. One patient (in the severe group) had elevation of all 3 indices ≥2 × ULN. The percentage of the patients with elevated both ALT and AST was 12.6% (10/79) in mild cases vs. 46.2% (12/26) in severe cases (*P* = 0.001). Nine patients (8.6%) had ≥3 × ULN elevation of ALT, AST or TBil.

### Categories analysis

Cases were divided into 4 categories based on ALT at admission and during hospitalization: Normal during the entire period, normal and then abnormal, abnormal and then normal, and abnormal during the entire period. The results are shown in Table 3. Overall, liver functions returned to normal range in 77 (73.3%) patients before discharge.

**Table 3:** ALT level change during hospitalization in patients with COVID-19 [n (%)]

Category	Overall (*n* = 105)	Mild group (*n* = 79)	Severe group (*n* = 26)	***P***-value
**Normal during the entire period**	68 (64.8)	56 (70.9)	12 (46.2)	0.032
**Normal and then abnormal**	20 (19.0)	11 (13.9)	9 (34.6)	0.007
**Abnormal and then normal**	9 (8.6)	7 (8.9)	2 (7.7)	1.000
**Abnormal during the entire period**	8 (7.6)	5 (6.3)	3 (11.5)	0.406

A total of 68 patients (64.8%, 68/105) had normal ALT during the entire period: 12 (46.2%, 12/26) in severe cases and 56 (70.9%, 56/79) in mild cases (*P* = 0.032).

Twenty (19.0%, 20/105) patients had normal ALT at admission but had elevated ALT during hospitalization: 9 (34.6%, 9/26) in severe cases and 11 (13.9%, 11/79) in mild cases (*P* = 0.007). Upon discharge from the hospital, ALT was still elevated in 10 mild cases (3 at > 2 × ULN) and in 2 severe cases (1 at > 2 × ULN, and 1 was died). Most of ALT elevation occurred between day 4 and day 17 of hospitalization, with a mean of 7.3 ± 3.0 d in severe cases vs. 10.7 ± 4.1 d in mild cases (*P* = 0.048). Excluding 1 patient with delayed detection for personal reasons, ALT assessment was conducted every 3.0 ± 0.9 d between admission and the onset of ALT elevations in mild cases vs. 2.1 ± 0.6 d in severe cases (*P = 0.*015).

ALT was elevated at admission but normalized during hospitalization in 9 (8.6%, 9/105) patients; 7 patients (8.9%, 7/79) in mild cases and 2 patients (7.7%, 2/26) in severe cases (*P* = 1.000). ALT was elevated at admission and remained elevated during hospitalization in 8 (7.6%, 8/105) patients: 5 (6.3%, 5/79) in mild cases and 3 (11.5%, 3/26) in severe cases (*P* = 0.406). ALT in the last test remained elevated in 5 patients.

### Dynamic ALT change in a representative case with mild illness

**Fig. 3:**
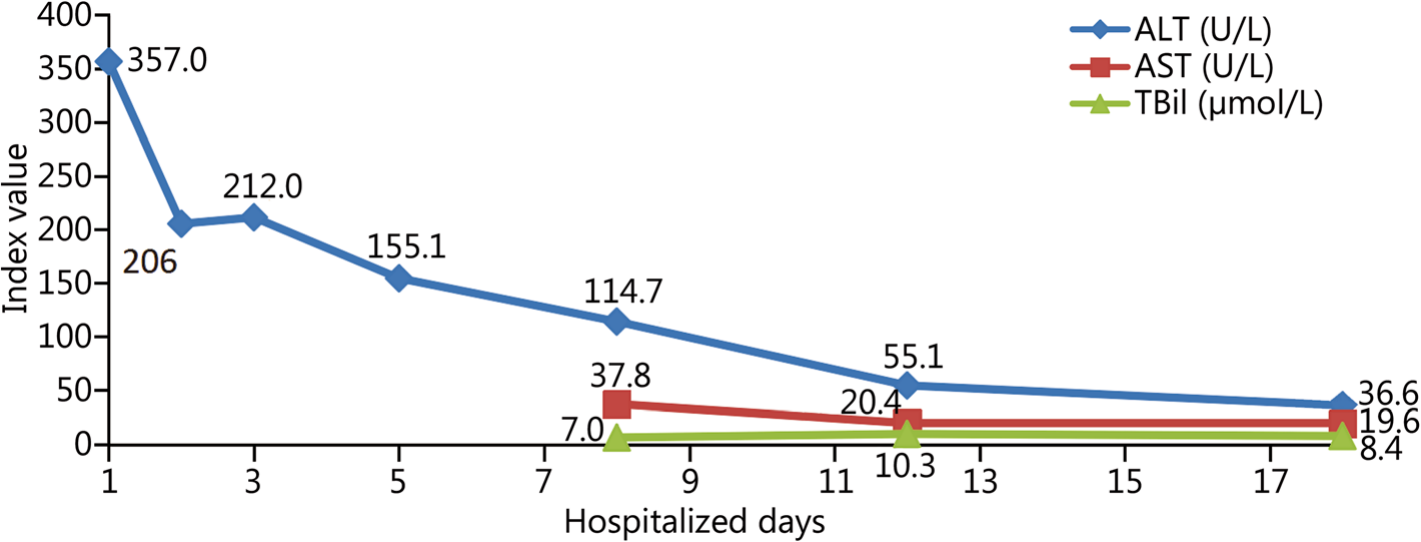
Dynamics of liver function indexes in 1 mild type patient with ALT elevation. A 45-year-old man has ALT > 7 × ULN (357.0 U/L) upon admission. He was admitted to the hospital on January 28, 2020 with a diagnosis of COVID-19. During the treatment, ALT gradually returned to normal. After 2 consecutive negative test for SARS-CoV-2 (on February 10, 2020 and February 12, 2020), he was discharged

A 45-year-old man has ALT > 7 × ULN (357.0 U/L) upon admission. He presented with cough, fever, and chills on January 24, 2020 and was admitted to the hospital on January 28, 2020 with a diagnosis of COVID-19. He denied heavy alcohol use and chronic liver disease. Abdominal ultrasound showed no fatty liver, and HBsAg and anti-HCV were negative. Treatments included antipyretic, nutritional support, recombinant human interferon α-2b, lopinavir ritonavir tablet, reduced glutathione and compound glycyrrhizin. During the treatment, ALT gradually returned to normal (Fig. 3). After 2 consecutive negative test for SARS-CoV-2 (on February 10, 2020 and February 12, 2020), he was discharged.

## Discussion

Consistent with the previous reports [2], patients with severe illness in the current study had older age, longer duration from disease onset to hospital admission, and higher rate of men was larger. Fatty liver was detected in 40.0% (26/65) of mild cases and 45.8% (11/24) of severe cases. Such a rate is comparable to that in the general population [7], suggesting that fatty liver is not a possible predisposing factor for liver injury in COVID-19 patients.

Liver biochemistry abnormality in COVID-19 patients could be attributed to a variety of factors, including direct hepatocyte injury by the virus [8], drug-induced liver injury [1, 9], hypoxic-ischemic microcirculation disorder, and underlying liver diseases. Temporal relationship is an important clue in the identification of direct injury vs. drug-induced liver injury, whereas hepatic injury associated with hypoxic-ischemic microcirculation disorder may be more common in critically ill patients. In the current study, 21.6% of the abnormal test occurred after hospital admission. AST is widely distributed in muscle, cardiac myocytes and mitochondria. As a result, AST alone is a poor indicator of liver injury. The percentage of patients with elevated ALT or TBil was < 20.0% at admission, but in severe cases the rate of elevated AST was 63.6%. During hospitalization, the percentage of the patients with elevated ALT, AST as well as TBil in severe cases was 26.9%, which was significantly higher than 1.3% that in mild cases. Consistent with exudative lesions in the lungs, poor nutritional intake after onset in COVID-19 patients, albumin reduction was more pronounced in severe cases than in mild cases. Whether liver injury contributed to albumin change remains unknown.

Further analysis showed that 56.2% of the patients had elevated ALT, AST, or TBil levels during the course of COVID-19, which was higher than the rate at admission. The percentage of elevated TBil during hospitalization was 20.8% in mild cases and 36.0% in severe cases; both were higher than previously reported (9.8 and 20.8%) [1], and we speculate that the reason for this phenomenon might be related to the higher proportion of severe group patients in this study. One patient had ALT as high as 7590 U/L in previous report without clear course [2], but the highest ALT in this study was only 357.0 U/L. The percentage of the patients with elevated ALT, AST as well as TBil was 7.6% in the current study. Only 1 patient who was critically ill had all 3 indices at ≥2 × ULN. These findings suggest that liver damage is common but generally mild in COVID-19 patients.

A pattern analysis of ALT change during the study period showed that majority (68 out of 105) of the patients had normal ALT during the entire course of disease. Eight patients had elevated ALT throughout the study period. Considering the mechanism of COVID-19 liver injury, we speculate that these patients might need additional relevant testing to identify underlying causes for liver biochemistry abnormality. Most of patients had ALT elevations between days 4 and 17 of hospitalization, with a mean of 7.3 d in severe cases and 10.7 d in mild cases. Since there was difference in testing frequency between the two groups, the difference (7.3 d vs. 10.7 d) could be partly reasonably attributed to a difference in disease severity. This finding is consistent with the notion that COVID-19 tend be mild in early stages, but worsens typically in one week [1]. ALT abnormalities in these patients require further exploration, such as drug-induced liver injury [9] or associated with changes of disease status.

This study had some limitations. First, it is a retrospective analysis of the data collected from a single center. Second, it has been postulated that novel coronaviruses can enter bile duct epithelial cells through angiotensin-converting enzyme 2 (ACE2) receptors to cause liver injury [10], but alkaline phosphatase (ALP) and γ-glutamyltransferase (GGT) have not been found to be elevated [11]. We did not assess ALP and GGT in the current study. Medication history in many patients was unknown. Last but not least, the impact of co-morbidities (i.e., hypertension, coronary heart disease, type 2 diabetes mellitus, hypothyroidism and chronic obstructive pulmonary disease) was not investigated.

## Conclusions

A significant proportion of patients with COVID-19 maintained normal liver function throughout the course of their disease, but patients with severe illness were more likely to have abnormal liver function. Some patients started to have abnormal liver function parameters during treatment after admission, but most patients had mild and isolated elevations in ALT, AST, or TBil. Most of the patients discharged with normal liver function, and further identification of the aetiology is required for those who did not recover.

## Data Availability

The datasets used and/or analyzed during the current study are available from the corresponding author on reasonable request.

## Abbreviations

ACE2: Angiotensin-converting enzyme 2
ALT: Alanine aminotransferase
AST: Aspartate aminotransferase
GGT: γ-glutamyltransferase.
TBil: Total bilirubin
ICU: Intensive Care Unit
PaO_2_: Partial arterial oxygen pressure
FiO_2_: Fraction of inspired oxygen
ALB: Albumin; CHE: Cholinesterase
ALP: Alkaline phosphatase
